# Serum sTWEAK and FGF-23 Levels in Hemodialysis and Renal Transplant Patients

**Published:** 2017-05-01

**Authors:** H. Eskandari Naji, A. Ghorbanihaghjo, H. Argani, S. Raeisi, J. Safa, A. H. Alirezaei, N. Rashtchizadeh

**Affiliations:** 1 Department of Clinical Biochemistry and Laboratory Medicine, Tabriz University of Medical Sciences, Tabriz, Iran; 2 Drug Applied Research Center, Tabriz University of Medical Sciences, Tabriz, Iran; 3 Department of Urology, Shahid Modarres Hospital, Urology and Nephrology Research Center, Shahid Beheshti University of Medical Sciences, Tehran, Iran; 4 Division of Clinical Laboratory, Children’s Hospital, Tabriz University of Medical Sciences, Tabriz, Iran; 5 Biotechnology Research Center, Tabriz University of Medical Sciences, Tabriz, Iran

**Keywords:** Renal dialysis, TL1 cytokine [Supplementary Concept], sTWEAK, Kidney transplantation, Fibroblast growth factor 23 [Supplementary Concept]

## Abstract

**Background:**

Kidney transplantation is the treatment of choice for patients with end-stage renal disease.

**Objective:**

To evaluate the changes in serum soluble TNF-like weak inducer of apoptosis (sTWEAK) and fibroblast growth factor 23 (FGF-23) in hemodialysis (HD) patients and renal transplant recipients (RTR).

**Methods:**

Serum samples were obtained from 30 patients on chronic HD, 30 RTRs, and 30 normal controls. Biochemical factors, sTWEAK, FGF-23, and interlukin-6 (IL-6) were measured by standard methods.

**Results:**

Serum levels of sTWEAK in RTRs were significantly higher than those in the HD patients (p=0.025); RTR and HD patients had significantly lower sTWEAK levels than the controls (p=0.001 and p= 0.038, respectively). Serum levels of FGF-23 in HD patients were significantly (p=0.001) higher than those in the RTR; the level was higher in both studied groups compared to that in the controls (p=0.001 for both groups). The mean serum level of IL-6 in HD was significantly higher than that in RTR patients (p=0.013). IL-6 levels in both groups were significantly higher than those in controls (p=0.001 and p= 0.012, respectively). In HD group a negative correlation was found between FGF-23 and sTWEAK (r= 0.375, p=0.041); there were also a significant correlation between FGF-23 and IL-6 (r= 0.480, p= 0.007) and between IL-6 and sTWEAK (r= 0.409, p=0.025).

**Conclusion:**

We found that serum sTWEAK is decreased and FGF-23 is increased in HD and RTR groups comparing with the control group. However, further studies are needed to shed light over their direct role on atherosclerosis and cardiovascular outcomes.

## INTRODUCTION

Chronic kidney disease (CKD) is a general clinical term referred to all heterogeneous structural and functional renal disorders. Based on the glomerular filtration rate (GFR), CKD is classified into five stages; the 5th stage is end-stage of renal disease (ESRD) [1]. To avoid life-threatening uremia, patients with ESRD should be either on chronic hemodialysis (HD) or receive renal transplantation (RT) [2, 3]. 

Cardiovascular disease (CVD) is believed the most common cause of death in patients with CKD [4]. Dyslipidemia, hypertension, diabetes mellitus, and premature atherosclerosis are major causes of CVD in CKD patients [5]. Endothelial dysfunction (ED) is the first pathophysiologic step in vascular damage and premature atherosclerosis that ends to clinical CVD [6]. One of the causes of ED is mediated by soluble TNF-like weak inducer of apoptosis (sTWEAK) [7]. This factor is recently known as a novel biomarker for ED in patients with CKD [8]. TWEAK is expressed and found mainly in heart, brain, pancreas, intestine, lung, ovary, liver, and kidney. TWEAK after binding to its receptor, fibroblast growth factor inducible 14 (fn14), can mediate cellular proliferation, migration, survival, differentiation, osteoclastogenesis, angiogenesis, and apoptosis [9]. Although TWEAK facilitates physiologic tissue repair and regenerates acute injury, the irregular expression of TWEAK in chronic inflammatory diseases can be pathogenic [10]. It was reported that TWEAK can activate the inflammatory response during kidney disease [11] that may have additive effects on mortality in HD patients [12]. 

Hyperphosphatemia is a risk factor for the development of various complications of CKD such as CVD because of the calcium-phosphate deposits formation. Fibroblast growth factor-23 (FGF-23), a bone-derived phosphaturic factor, has important role as a hormonal regulator of phosphate homeostasis [13]. It was shown that high serum levels of FGF-23 in CKD patients are linked to increased mortality rates and vascular calcification [14]. FGF-23 may also have vascular toxicity [15]. It has been reported that serum FGF-23 level is a predictor of artery calcification in patients undergoing HD [15, 16]. Elevated levels of FGF-23 were also shown in patients after kidney transplantation, even with normal graft function [17]. As inflammation is the main cause of CVD in CKD patients, including HD and RTR, we conducted this study to measure FGF-23 and sTWEAK levels in HD patients and RTRs.

## PATIENTS AND METHODS

This study was performed in Drug Applied Research Center, Tabriz University of Medical Sciences (TUMS), Tabriz, Iran. It was approved by Tehran University of Medical Sciences Ethics Committee (Code: 5/79/281). Patients recruited to the study from December 2013 to April 2014. Thirty HD patients (15 men and 15 women) and 30 age-matched RTRs (18 men and 12 women) were included in this study. Written informed consent was obtained from all patients. Exclusion criteria were consumption of antioxidants, active infections, heart failure, malignancy, liver diseases, and severe anemia (Hb<10 g/dL). All HD patients were received rh-erythropoietin. Inclusion criteria in the RTR included receiving triple immunosuppressive drugs composed of cyclosporine (Zahravi Co.), CellCept (Roch Co.), and prednisolone (Abidi Co.); and absence of acute allograft rejection during the last three months. All patients in the HD and RTR groups received 1, 25(OH)_2_-D_3_ (Calcitiol; Roch Co.) supplementation 0.25 mg/day. The causes of renal failure in these patients were diabetic nephropathy, chronic glomerulonephritis, polycystic kidney disease, hypertensive ischemic nephropathy, obstructive nephropathy, and unknown etiology.

All of the HD patients were stable and under regular hemodialysis for more than 14 (range: 14–47) months, 3×4 hrs/wk by synthetic high-flux membranes with Fresenius-2008 B hemodialyser. Thirty age- and sex-matched healthy individuals (15 men, 15 women) served as the control group. They were subjected to the same inclusion and exclusion criteria as the HD and RTR patients. All samples were obtained from the peripheral vein after 12-hr of overnight fasting. Measurement were done in the HD patients just prior to the beginning of hemodialysis and in the RTR group prior to consumption of the next immunosuppressive drugs (for through level of cyclosporine). Sera were separated within 30 min and sampling and were kept frozen at 70 °C until analyses were done (maximum of 5 months). Serum creatinine, albumin, and urea levels were measured by enzymatic colorimetric methods with an automated chemical analyzer (Abbott analyzer, Abbott laboratories, Abbott Park, North Chicago, IL). Serum total calcium and phosphorus were measured by commercial kits (Pars Azmoon Co, Iran). Serum concentrations of TWEAK was measured by human TWEAK enzyme-linked immunosorbent assay (ELISA) kit (Bioassay technology lab, China, Catalog No: E1820HU20140926) by an ELISA plate reader (STATFAX-2100, Multi-detection Multi Plate Reader, USA). The detection range of TWEAK ELISA kit was 10–4000 ng/mL. FGF-23 concentration was also determined by ELISA (Bioassay technology lab, China, Catalog No: E0059HU20140926) with 5–1500 pg/mL detection range. Interleukin-6 (IL-6) was measured by ELISA too (DIA source, Belgium, catalog number: KAP1261) with a detection range of 0–50 pg/mL.

Statistical Analysis

Statistical analysis was performed by SPSS^®^ ver 18 for Windows^®^. Values were expressed as median (range) for nonparametric and mean±SD for parametric data. Numbers and their percentages were presented when appropriate. Differences among groups were assessed by Mann-Whitney U test for the nonparametric data or one-way ANOVA for parametric data. Spearman’s *ρ* was calculated to determine the correlation between the parameters. A p value <0.05 was considered statistically significant.

## RESULTS

Table 1 shows demographic characteristics of the 30 HD patients, 30 RTRs, and 30 normal controls. There were no significant differences in the mean age and sex distribution among the three groups. The mean serum level of calcium in RTR and control groups was significantly (p<0.001) higher than that in HD group; however, the mean phosphorus level in HD group was significantly higher than that in RTR and control groups. The median serum level of IL-6 in HD group was higher than that in RTRs (29.1 [range: 5.6–380.0] vs. 16.5 [4.2–318.0] pg/mL; p=0.013). The median serum sTWEAK level in RTRs was significantly higher than that in the HD group (734.50 [168.9–2546.0] vs. 598.8 [115.2–1271.0] ng/mL; p=0.025]. In addition, both HD and RTR groups had significantly lower sTWEAK levels than the control group (p=0.001 and p=0.038, respectively]—sTWEAK level: controls>RTRs>HD patients**.**

**Table 1:** Demographic data for the hemodialysis, renal transplant recipients, and normal group

Variable	Control group (n=30)	HD group (n=30)	RTR group (n=30)	p value^c^
Age (yr) (mean±SD)	50.4±3.6	50.7±5.5 p^a^=0.782	48.5±5.5 p^b^=0.124	p^c^=0.124
Sex (male/female)	15/15	15/15 p^a^=1.000	18/12 p^b^=0.440	p^c^=0.440
Underlying disorders; n (%)				
Diabetic nephropathy	—	10 (33%)	15 (50%)	—
Chronic glomerulonephritis	—	1 (3%)	2 (7%)	—
Polycystic kidney disease	—	6 (20%)	2 (7%)	—
Hypertensive ischemic nephropathy	—	8 (27%)	6 (20%)	—
Obstructive nephropathy	—	3 (10%)	5 (17%)	—
Unknown etiology	—	2 (7%)	0 (0%)	—
Duration of dialysis (month) (mean±SD)	—	31.03±9.34	19.57±4.68 (before RT)	p^c^=0.001
Conventional therapy (%)			—	—
CaCO_3_	—	73%	—	—
Venofer	—	77%	—	—
Heparin	—	100%	—	—
Erythropoietin	—	100%	—	—
Cyclosporine	—	—	100%	—
Prednisolone	—	—	100%	—
CellCept	—	—	100%	—

a Comparison of HD group *vs*. Control group

b Comparison of RTR group *vs.* Control group

c Comparison of HD group *vs*. RTR group

The median serum FGF-23 level in HD patients was significantly higher than that in the RTRs (460.7 [269.2–2326.0] vs. 297.3 [200.0–1494.0] pg/mL; p<0.01); both of these groups, however, had significantly (p<0.05) higher FGF-23 levels compared to the control group (236.4 [86.9–422.50] pg/mL; p<0.01 vs. HD, p<0.01 vs. RTR)—FGF-23 level: HD patients>RTRs>controls (Table 2).

**Table 2:** Comparison of lab results among three studied groups

Variable	Control group (n=30)	HD group (n=30)	RTR group (n=30)	p value^c^
Calcium (mg/dL) (mean±SD)	9.03±0.65	7.97±1.12 p^a^=0.001	9.18±0.97 p^b^= 0.135	p=0.001
Phosphorus (mg/dL) (mean±SD)	3.76±0.462	5.96±0.689 p^a^=0.001	3.57±0.683 p^b^=0.248	p=0.001
Creatinine (mg/dL) [median (min–max)]	1.0 (0.7-1.30)	7.0 (3.50-10.40) p^a^=0.001	1.7 (0.80-6.10) p^b^<0.001	p=0.001
Urea (mg/dL) (mean±SD)	28.3±12.28	112.5±27.81 p^a^=0.001	40.35±9.45 p^b^=0.001	p=0.001
Albumin (g/dL) (mean±SD)	4.88±0.22	3.75±0.51 p^a^=0.001	4.46±0.47 p^b^=0.001	p=0.001
sTWEAK (ng/mL) [median (min–max)]	893.5 (508.6–3950.0)	598.8 (115.2–1271.0) p^a^=0.001	734.5 (168.9–2546.0) p^b^=0.038	p=0.025
FGF-23 (pg/mL) [median (min–max)]	236.4 (86.9–422.5)	460.7 (269.2–2326.0) p^a^=0.001	297.3 (200.0–1494.0) p^b^=0.001	p=0.001
IL-6 (pg/mL) [median (min–max)]	9.3 (2.1–97.2)	29.150 (5.6–380.0) p^a^=0.001	16.5 (4.2–318.0) p^b^=0.012	p=0.013

a Comparison of HD group *vs*. Control group

b Comparison of RTR group *vs.* Control group

c Comparison of HD group *vs*. RTR group

The correlations of sTWEAK, FGF-23, and IL-6 in HD group are shown in Table 3. A negative correlation was found between FGF-23 and sTWEAK (r= 0.375, p=0.041) in HD group. Also in HD group were significant correlations between FGF-23 and IL-6 (r= 0.480, p= 0.007), and IL-6 and sTWEAK (r= 0.409, p=0.025). There were no other significant correlations among other studied groups.

**Table 3:** The correlations of sTWEAK, FGF-23 and IL-6 in HD group

Patient group	FGF-23 and sTWEAK		FGF-23 and IL-6		sTWEAK and IL-6	
	r	p	r	p	r	p
HD	0.375	0.041	0.480	0.007	0.409	0.025

## DISCUSSION

The aim of present study was to evaluate the changes in serum levels of sTWEAK and FGF-23 in HD and RTR patients and compare the results with a healthy control group. sTWEAK was recently introduced as a TNF-related cytokine in various inflammatory disorders such as CKD [18]. Inflammation in CKD patients ends to cardiovascular morbidity and mortality [19, 20]. Meier, *et al*, reported that sTWEAK plasma level decreases with impaired renal function in ESRD patients and is also associated with their mortality risk [21]. In our study, serum levels of sTWEAK in ESRD patients (HD and RTR groups) were significantly lower than those in the controls. These results were in agreement with previous studies such as Hassan Seham, *et al* [7], Yilmaz, *et al* [8], Carrero, *et al* [12], Kralisch, *et al* [22], and Turkmen, *et al* [23]. Also in our study, similar to the study of Turkmen, *et al*. [23], HD patients had significantly lower sTWEAK level compared RTRs. Decreased levels of serum sTWEAK in these patients might be associated with ongoing inflammation in this population [23]. Recently, fn14 and also CD163, a scavenger receptor, were introduced as receptors of sTWEAK [9, 24]. Binding of sTWEAK to fn14 activates the I kappa kinase (IKK) complex. This activation is the signaling pathway of nuclear factor kappa-light-chain enhancer of activated B cells (NFKβ) [23, 25] that mediates multiple effects such as inflammation [26]. Moreno, *et al* [27], in an animal model, and Muñoz-García, *et al* [10], in humans, showed that the expression of fn14 is increased under pathologic conditions. According to the study of Winkles, *et al* [28], under inflammatory conditions any change in serum levels of sTWEAK may due to fn14 overexpression. Activation of NFKβ, because of TWEAK-fn14 interaction, can upregulate the expression of inflammatory cytokines such as IL-6. It can be supposed that increased IL-6 can upregulate the expression of fn14 causing a vicious cycle that may potentiate the association of sTWEAK with mortality [12]. We found that serum IL-6 levels were significantly lower in the controls in comparison with each of HD and RTR groups. Carrero, *et al* [12], reported that sTWEAK is negatively associated with IL-6, the same result we found in our study. Recently, Du, *et al* [29], showed that cyclosporine A (CsA) can lead to NFKβ down-regulation in renal tubular cells. We found that serum level of IL-6 in RTR group was lower than that in HD group. Based on the study of Du, *et al* [29], this result may be attributed to the effect that CsA is administered as an immunosuppressive drug to RTR group.

CKD patients have impaired renal excretion of phosphate leading to hyperphosphatemia [30] that is the reason of several complications such as formation of ectopic calcifications and CVD [31]. FGF-23 with its co**-**receptor, klotho, acts as a phosphatonin inhibiting renal phosphate reabsorption [32, 33]. Increased serum levels of FGF-23 in renal failure could also be due to a direct physiologic response to hyperphosphatemia [13]. According to previous studies, FGF-23 level is associated with vascular calcification and increased mortality in CKD patients [34, 35]. Desjardins, *et al*, and other studies suggest that serum FGF-23 level can be an independent biomarker of vascular calcification in CKD patients [14-16, 36]. In our study, FGF-23 levels in HD patients were significantly higher than those in RTR group. It is probably due to high levels of phosphorus and low levels of calcium in the HD patients in order to regulate these minerals. Although the main physiological role of FGF-23 is to maintain a stable serum phosphate levels, Marsell, *et al* [37], and Roos, *et al* [38], in their study reported that there is no correlation between FGF-23 and phosphate concentrations in subjects with normal kidney function. Moreover, Torres, *et al*, showed the same results in HD patients [39]. We also found no significant correlation between FGF-23 with either phosphorus or calcium levels in HD and RTR patients. 

As FGF-23 concentration reflects phosphorus accumulation in CKD patients [13], and because hyperphosphatemia increases significantly the CVD risk [40, 41], FGF-23 may be a better predictor of CKD progression (time to doubling of serum creatinine) than calcium or phosphate levels. 

Mendoza, *et al* [42], in their study showed that higher FGF23 levels are independently associated with higher levels of inflammatory markers in patients with CKD. We also found a significant correlation between FGF-23 and IL-6 in HD group. Our results in HD group showed that there was a negative correlation between FGF-23 and sTWEAK. Moreno, *et al* [43], in their study, showed that inflammatory cytokines, such as TWEAK and TNFα could down regulate klotho expression through an NFKβ-dependent mechanism in cultured tubular cells and in the kidney *in vivo*. These results may explain the relationship between inflammation and diseases in CKD patients. Considering that klotho is co**-**receptor of FGF-23, its down-regulation by TWEAK may lead to down-regulation of FGF-23, as a compensatory mechanism. If the theory is confirmed by future studies, it would be a reasonable response to the negative correlation between FGF-23 and sTWEAK in HD group observed in our study.

Our study had some limitations: for its cross-sectional nature, we could not determine the changes in serum sTWEAK, FGF-23, and IL-6 levels; we could not also evaluate their effects on prognosis and follow-up the patients. Moreover, the sample size of our study was relatively small.

## References

[1] Levey A, Coresh J (2012). Chronic kidney disease. The Lancet.

[2] Eryilmaz M, Ozdemir C, Yurtman F (2005). Quality of sleep and quality of life in renal transplantation patients. Transplant proc.

[3] Sayin A, Mutluay R, Sindel S (2007). Quality of life in hemodialysis, peritoneal dialysis, and transplantation patients. Transplant Proc.

[4] Collins AJ (2003). Cardiovascular mortality in end-stage renal disease. Am J Med Sci.

[5] Dasmahapatra P, Srinivasan SR, Mokha J (2011). Subclinical atherosclerotic changes related to chronic kidney disease in asymptomatic black and white young adults: the Bogalusa Heart Study. Ann Epidemiol.

[6] Libby P, Ridker PM, Maseri A (2002). Inflammation and atherosclerosis. Circulation.

[7] Hassan SB, El-demery AB, Ahmed AI, Abukhalil RE (2012). Soluble TWEAK and cardiovascular morbidity and mortality in chronic kidney disease patients. Arab J Nephrol Transplant.

[8] Yilmaz MI, Carrero JJ, Ortiz A (2009). Soluble TWEAK plasma levels as a novel biomarker of endothelial function in patients with chronic kidney disease. Clin J Am Soc Nephrol.

[9] Polek TC, Talpaz M, Darnay BG, Spivak-Kroizman T (2003). TWEAK mediates signal transduction and differentiation of RAW264.7 cells in the absence of Fn14/TweakR. Evidence for a second TWEAK receptor. J Biol Chem.

[10] Muñoz-García B, Martín-Ventura JL, Martínez E (2006). Fn14 is upregulated in cytokine-stimulated vascular smooth muscle cells and is expressed in human carotid atherosclerotic plaques modulation by atorvastatin. Stroke.

[11] Sanz AB, Justo P, Sanchez-Niño MD (2008). The cytokine TWEAK modulates renal tubulointerstitial inflammation. J Am Soc Nephrol.

[12] Carrero JJ, Ortiz A, Qureshi AR (2009). Additive effects of soluble TWEAK and inflammation on mortality in hemodialysis patients. Clin J Am Soc Nephrol.

[13] Larsson T, Nisbeth U, Ljunggren Ö (2003). Circulating concentration of FGF-23 increases as renal function declines in patients with chronic kidney disease, but does not change in response to variation in phosphate intake in healthy volunteers. Kidney international.

[14] Jean G, Terrat J-C, Vanel T (2009). High levels of serum fibroblast growth factors (FGF)-23 are associated with increased mortality in long haemodialysis patients. Nephrol Dial Transplant.

[15] Ashikaga E, Honda H, Suzuki H (2010). Impact of fibroblast growth factor 23 on lipids and atherosclerosis in hemodialysis patients. Ther Apher Dial.

[16] Nasrallah M, El-Shehaby A, Salem M (2010). Fibroblast growth factor -23 (FGF-23) is independently correlated to aortic calcification in haemodialysis patients. Nephrol Dial Transplant.

[17] Evenepoel P, Naesens M, Claes K (2007). Tertiary ‘hyperphosphatoninism’accentuates hypophosphatemia and suppresses calcitriol levels in renal transplant recipients. Am J Transplant.

[18] Burkly LC, Michaelson JS, Zheng TS (2011). TWEAK/Fn14 pathway: an immunological switch for shaping tissue responses. Immunol Rev.

[19] Ahmed M, Wong C, Pai P (2010). Cardiovascularsyndrome : a new classification and current evidence on its management. Clin Nephrol.

[20] Sarnak MJ (2003). Cardiovascular complications in chronic kidney disease. Am J Kidney Dis.

[21] Meier P (2011). Plasma sTWEAK and PTX3: New determinant tools of cardiovascular outcome also in patients with CKD. Clin J Am Soc Nephrol.

[22] Kralisch S, Ziegelmeier M, Bachmann A (2008). Serum levels of the atherosclerosis biomarker sTWEAK are decreased in type 2 diabetes and end-stage renal disease. Atherosclerosis.

[23] Turkmen K, Tonbul HZ, Erdur FM (2013). Soluble TWEAK independently predicts atherosclerosis in renal transplant patients. BMC nephrology.

[24] Wiley SR, Winkles JA (2003). TWEAK, a member of the TNF superfamily, is a multifunctional cytokine that binds the TweakR/Fn14 receptor. Cytokine growth factor rev.

[25] Roos C, Wicovsky A, Müller N (2010). Soluble and transmembrane TNF-like weak inducer of apoptosis differentially activate the classical and noncanonical NF-kappa B pathway. J Immunol.

[26] Ortiz A, Sanz AB, García BM (2009). Considering TWEAK as a target for therapy in renal and vascular injury. Cytokine Growth Factor Rev.

[27] Moreno JA, Dejouvencel T, Labreuche J (2010). Peripheral artery disease is associated with a high CD163/TWEAK plasma ratio. Arterioscler Thromb Vasc Biol.

[28] Winkles JA (2008). The TWEAK–Fn14 cytokine–receptor axis: discovery, biology and therapeutic targeting. Nat Rev Drug Discov.

[29] Du S, Hiramatsu N, Hayakawa K (2009). Suppression of NF-kappaB by cyclosporin a and tacrolimus (FK506) via induction of the C/EBP family: implication for unfolded protein response. J Immunol.

[30] Locatelli F, Cannata-Andía JB, Drüeke TB (2002). Management of disturbances of calcium and phosphate metabolism in chronic renal insufficiency, with emphasis on the control of hyperphosphataemia. Nephrol Dial Transplant.

[31] Qunibi WY, Nolan CA, Ayus JC (2002). Cardiovascular calcification in patients with end-stage renal disease: a century-old phenomenon. Kidney international.

[32] Baum M, Schiavi S, Dwarakanath V, Quigley R (2005). Effect of fibroblast growth factor-23 on phosphate transport in proximal tubules. Kidney international.

[33] Gattineni J, Bates C, Twombley K (2009). FGF23 decreases renal NaPi-2a and NaPi-2c expression and induces hypophosphatemia in vivo predominantly via FGF receptor 1. Am J Physiol Renal Physiol.

[34] Kocełak P, Olszanecka-Glinianowicz M, Chudek J (2012). Fibroblast growth factor 23—structure, function and role in kidney diseases. Adv Clin Exp Med.

[35] Yasin A, Liu D, Chau L (2013). Fibroblast growth factor-23 and calcium phosphate product in young chronic kidney disease patients: a cross-sectional study. BMC nephrology.

[36] Desjardins L, Liabeuf S, Renard C (2012). FGF23 is independently associated with vascular calcification but not bone mineral density in patients at various CKD stages. Osteoporos Int.

[37] Marsell R, Grundberg E, Krajisnik T (2008). Fibroblast growth factor-23 is associated with parathyroid hormone and renal function in a population-based cohort of elderly men. Eur J Endocrinol.

[38] Roos M, Lutz J, Salmhofer H (2008). Relation between plasma fibroblast growth factor-23, serum fetuin-A levels and coronary artery calcification evaluated by multislice computed tomography in patients with normal kidney function. Clin Endocrinol (Oxf).

[39] Torres PU, Friedlander G, De Vernejoul M (2008). Bone mass does not correlate with the serum fibroblast growth factor 23 in hemodialysis patients. Kidney Int.

[40] Block GA, Klassen PS, Lazarus JM (2004). Mineral metabolism, mortality, and morbidity in maintenance hemodialysis. J Am Soc Nephrol.

[41] Kalantar-Zadeh K, Kuwae N, Regidor D (2006). Survival predictability of time-varying indicators of bone disease in maintenance hemodialysis patients. Kidney Int.

[42] Munoz Mendoza J, Isakova T, Ricardo AC (2012). Fibroblast growth factor 23 and Inflammation in CKD. Clin J Am Soc Nephrol.

[43] Moreno JA, Izquierdo MC, Sanchez-Nino MD (2011). The inflammatory cytokines TWEAK and TNFalpha reduce renal klotho expression through NFkappaB. J Am Soc Nephrol.

